# Fetal and Maternal Outcomes of Planned Pregnancy in Patients with Systemic Lupus Erythematosus: A Retrospective Multicenter Study

**DOI:** 10.1155/2018/2413637

**Published:** 2018-09-03

**Authors:** Dongying Chen, Minxi Lao, Jianyu Zhang, Yanfeng Zhan, Weinian Li, Xiaoyan Cai, Zhongping Zhan

**Affiliations:** ^1^Department of Rheumatology, The First Affiliated Hospital of Sun Yat-sen University, No. 58, Zhongshan 2nd Road, Guangzhou 510080, China; ^2^Department of Rheumatology and Department of Geriatrics, The First Affiliated Hospital of Sun Yat-sen University, No. 58, Zhongshan 2nd Road, Guangzhou 510080, China; ^3^Department of Rheumatology, The Third Affiliated Hospital of Guangzhou Medical University, No. 63, Duobao Road, Guangzhou 510150, China; ^4^Department of Obstetrics and Gynecology, The First Affiliated Hospital of Sun Yat-sen University, No. 58, Zhongshan 2nd Road, Guangzhou 510080, China; ^5^Department of Rheumatology, Guangzhou First People's Hospital, The Second Affiliated Hospital of South China University of Technology, No. 1, Panfu Road, Guangzhou 510180, China

## Abstract

**Objective:**

To investigate the fetal and maternal outcomes as well as predictors of APOs in women with SLE who conceived when the disease was stable, the so-called “planned pregnancy.” *Methods*. A retrospective multicenter study of 243 patients with SLE who underwent a planned pregnancy was performed. APOs in fetus and mothers were recorded.

**Results:**

The average age at conception was 28.9 ± 3.9 years. Duration of SLE prior to pregnancy was 4.4 ± 4.3 years. Fetal APOs occurred in 86 (86/243, 35.4%) patients. Preterm births, intrauterine growth retardation (IUGR), fetal distress, and fetal loss accounted for 22.2%, 14.8%, 11.1%, and 4.9%, respectively. Forty-two preterm infants (42/54, 77.8%) were delivered after the 34th week of gestation. All the preterm infants were viable. Fifty-two patients (52/243, 21.4%) had disease flares, among which 45 cases (45/52, 86.5%) were mild, 6 (6/52, 11.5%) were moderate, and 1 (1/52, 1.9%) was severe. Disease flares were mainly presented as active lupus nephritis (41/52, 78.8%), thrombocytopenia (10/52, 19.2%), and skin/mucosa lesions (9/52, 17.3%). Pregnancy-induced hypertension (PIH) occurred in 29 patients, among which 3 were gestational hypertension and 26 were preeclampsia. Multiple analysis showed that disease flares (OR, 8.1; CI, 3.8–17.2) and anticardiolipin antibody positivity (OR, 7.4; CI, 2.5–21.8) were associated with composite fetal APOs.

**Conclusion:**

Planned pregnancy improved fetal and maternal outcomes, presenting as a lower rate of fetal loss, more favorable outcomes for preterm infants, and less severe disease flares during pregnancy.

## 1. Introduction

Compared with the general population, patients with SLE are still at high risk of adverse pregnancy outcomes (APOs) [1]. A number of studies have shown that patients with SLE are more likely to develop fetal complications, including fetal loss, preterm birth, and IUGR, compared to healthy women [2]. However, recent studies have reported that fetal outcomes are relatively favorable if lupus is stable or mildly active [3]. Moreover, the occurrence of disease flares increases during SLE pregnancies but decreases if pregnancy is delayed until disease is quiescent [4]. Currently, patients with SLE were advised to consider pregnancy during periods of inactive or stable disease [5], the so-called “planned pregnancy.” However, such researches usually enrolled a small number of candidates, and data is lacking in China.

Therefore, we performed a retrospective multicenter research in South China aimed at investigating the fetal and maternal outcomes, predictors of APOs, and pregnancy-associated disease flares in women with SLE who underwent planned pregnancy and close pregnancy monitoring by a multidisciplinary team.

## 2. Patients and Methods

### 2.1. Patient Population

A total of 243 patients with SLE who underwent planned pregnancy from three tertiary hospitals in Guangzhou from December 2011 to December 2016 were included (127 pregnancies in the First Affiliated Hospital of Sun Yat-sen University; 66 pregnancies in Guangzhou First People's Hospital, the Second Affiliated Hospital of South China University of Technology; and 50 pregnancies in the Third Affiliated Hospital of Guangzhou Medical University). Only one pregnancy for each patient was included. All patients fulfilled the 1997 ACR diagnostic criteria for SLE [6]. Planned pregnancy was defined according to Chinese recommendations for perinatal management in women with SLE [7], as lupus patients who were allowed to conceive under the situation of (1) stable disease activity for at least six months; (2) dose of oral prednisone < 15 mg per day); (3) urine protein < 0.5 g/24 hours; (4) absence of major organ dysfunction; (5) discontinuation of immunosuppressants including cyclophosphamide, methotrexate, and mycophenolate mofetil for at least six months; and (6) for those who were taking leflunomide, wash-out therapy should be administered and leflunomide was withdrawn for at least six months.

### 2.2. Screening and Follow-Ups

All pregnant women with SLE in the Chinese tertiary hospitals were followed according to Chinese recommendations for perinatal care in high-risk women [8], namely, every four weeks up to the 28th week of gestation and every two weeks from the 28th week up to delivery. Patients who were not followed regularly or without complete records were excluded. Regular obstetric practice, blood pressure, fetal heartbeat, clinical symptoms of lupus, laboratory tests including complete blood count, routine urine test, blood biochemical test, 24-hour urine protein, complement C3 and C4, anti-dsDNA antibodies, anti-SSA antibodies, anti-SSB antibodies, antiphospholipid antibody (including anticardiolipin (aCL) antibody IgM, aCL antibody IgG, and anti-*β*2GP1 antibody lupus anticoagulants (LAC)), and medical treatments were recorded. SLE activity was measured by the Systemic Lupus Erythematosus Pregnancy Disease Activity Index (SLEPDAI) at the first trimester, second trimester, and third trimester. The highest score was used in statistical analysis. Fetal Doppler sonography was performed every 4~8 weeks since the 16th week of gestation and every 2~4 weeks since the 28th week of gestation. Patients with positive antiphospholipid antibody were pretreated with aspirin. Patients with antiphospholipid antibody syndrome (APS) were pretreated with aspirin and low molecular heparin until delivery.

### 2.3. APOs

Fetal APOs include the following: (1) fetal loss, including spontaneous abortion (termination of pregnancy before the 20th week of gestation caused by natural factors), therapeutic abortion (artificial termination of pregnancy because of life-threatening progression of lupus or obstetric complications), stillbirth (intrauterine fetal demise after 20 weeks of gestation unexplained by chromosomal abnormalities, anatomic malformation, or congenital infection), and neonatal death referred to as the death of a live infant within 28 days after birth; (2) preterm birth (live birth before 37 weeks of gestation); (3) IUGR (birth weight below the 10th percentile of the Chinese population according to gestational week at delivery and fetal gender; and (4) fetal distress referred to as fetus hypoxia and acidosis, which could endanger the health of the fetus. Composite APOs were defined as the occurrence of any adverse outcomes including fetal loss, preterm birth, SGA babies, and fetal distress during gestation. Maternal APOs include the following: (1) disease flare was defined according to the International Consensus for disease flare in lupus [9], namely, new onset or worsening of specific and associated cutaneous manifestations of SLE; arthritis; one or more hemocytopenia not attributed to immunosuppressive drugs; neurological, cardiopulmonary, and renal manifestations; elevated serum creatinine in association with low-serum complement; and/or elevated titers of anti-dsDNA antibodies. Active lupus nephritis was defined according to the following: proteinuria > 0.5 g/24 h, active urinary sediment (>3 red blood cells/high-power field (HPF), or >5 white blood cells/HPF, or cellular casts), or estimated creatinine clearance (CrCl) <60 ml/min/1.73 m^2^ with active urinary sediment. Mild disease activity was defined as SLEPDAI score 5 to 9, moderate disease activity as SLEPDAI score 10 to 14, and severe disease activity as SLEPDAI score ≥ 15. (2) Pregnancy-induced hypertension (PIH) was defined as hypertension during pregnancy, which included gestational hypertension, preeclampsia, and eclampsia. Hypertension was defined as systolic blood pressure > 140 mmHg and/or diastolic blood pressure > 90 mmHg in sitting position in at least two consecutive measurements during pregnancy. Preeclampsia was defined as a new onset of hypertension with or without proteinuria after the 20th week of gestation in a previously normotensive woman [10].

### 2.4. Statistical Analysis

Software SPSS 20.0 was used for data analysis. Quantitative variables were recorded as mean ± standard deviation and compared by Student's *t*-test. Categorical variables were described as frequency and percentage and compared by a chi-square test. Factors related to APOs at *P* < 0.10 in univariate analyses were entered into a multivariate logistic model. *P* < 0.05 was considered as statistically significant.

### 2.5. Ethics Statement

Since this is a retrospective study, patient's interest was not involved; therefore, no ethical approval was required. The Ethical Committees of the above three centers waived that the research could be done based on a record review without contacting the patients. A support letter was obtained from the medical director's office of the three hospitals for retrieving retrospective data from the database and records. All the information was kept confidential, and no individual identifiers were collected.

## 3. Results

### 3.1. Demographic Data and History of Pregnancy

The average age at conception was 28.9 ± 3.9 years (20 to 38 years). Duration of SLE before pregnancy was 4.4 ± 4.3 years (1.5 to 21 years). Of the 243 patients, 146 patients became pregnant for the first time and 97 patients had prior history of pregnancy. Fifty-one patients had a history of adverse pregnancy, indicated abortion (*n* = 25), stillbirth (*n* = 3), premature delivery (*n* = 17), IUGR (*n* = 5), fetal distress (*n* = 1), and PIH (*n* = 5). Five patients experienced adverse pregnancy twice.

### 3.2. Fetal Outcomes

One hundred and fifty-seven (64.6%) patients ended in delivery without APOs, and 86 (35.4%) patients had at least one episode of APOs. Fetal APOs were shown in Table 1. In total, 12 patients (4.9%) experienced fetal loss. Spontaneous abortion, stillbirth, and therapeutic abortion accounted for one (0.4%), six (2.5%), and five (2.1%) cases, respectively. Causes of therapeutic abortion included active lupus nephritis (*n* = 3), severe thrombocytopenia (*n* = 1), and anatomic malformation (*n* = 1). Live birth delivery was succeeded in 231 patients (95.1%), among which 177 patients (72.8%) had term births, 54 (22.2%) had preterm births, 36 (14.8%) had IUGR, and 27 (11.1%) had fetal distress. Average birth weight was 2713.7 ± 521.3 g (790.0~4150.0 g). The average weights of preterm birth and term birth were 2198.5 ± 637.1 g (790.0~3200.0 g) and 2868.8 ± 359.0 g (1940.0~4150.0 g), respectively. Forty-two preterm infants (42/54, 77.8%) were delivered after the 34th week of gestation. Causes of preterm births included therapeutic preterm births (23/54, 42.6%), preterm premature rupture of membranes (PPROM) (19/54, 35.2%), and spontaneous preterm births (12/54, 22.2%). Preeclampsia (*n* = 14), lupus flares (*n* = 6), IUGR (*n* = 6), placenta previa (*n* = 3), and placental abruption (*n* = 2) were the main causes that led to therapeutic preterm births. All the preterm infants were viable.

### 3.3. Maternal Outcomes

Maternal APOs were presented in Table 1. Fifty-two disease flares (21.4%) occurred, among which 8 disease flares occurred during the first trimester, 15 during the second trimester, and 29 during the third trimester. Disease activity was mild in 45 (45/52, 86.5%) patients, moderate in 6 (6/52, 11.5%), and high in 1 (1/52, 1.9%). Disease flares were presented as active lupus nephritis (41/52, 78.8%), thrombocytopenia (10/52, 19.2%), skin/mucosa lesions (9/52, 17.3%), leukopenia (6/52, 11.5%), arthritis (6/52, 11.5%), alopecia (2/52, 3.8%), hemolytic anemia (1/52, 1.9%), and pulmonary hypertension (1/52, 1.9%). All disease flares were promptly diagnosed and treated when necessary. Of the 52 flares, 20 were treated with an increase of oral prednisone, associated with three intravenous methylprednisolone pulses. Prednisone was prescribed 10 to 15 mg per day to patients with mild disease activity, 15 to 30 mg per day to patients with moderate disease activity, and over 30 mg per day to patients with high disease activity. In total, 27 patients (11.1%) took prednisone more than 10 mg per day. Increased azathioprine dose was prescribed to three patient, and introduction of azathioprine to seven cases. One patient with severe disease flares received therapeutic abortion and was treated with cyclophosphamide subsequently. Of these 52 pregnancies, 27 ended in preterm births, 16 in IUGR, 9 in fetal distress, and 11 in fetal loss. Causes for fetal loss in patients with disease flare included therapeutic abortion (*n* = 5, 9.6%), spontaneous abortion (*n* = 1, 1.9%), and stillbirth (*n* = 5, 9.6%).

In this study, PIH occurred in 29 cases, among which 3 were gestational hypertension and 26 were preeclampsia. No eclampsia occurred. Of the 29 patients, fetal loss occurred in 5 patients, preterm births in 20, IUGR in 13, and fetal distress in 10. Causes for fetal loss in patients with PIH included therapeutic abortion (*n* = 2, 6.9%) and stillbirth (*n* = 3, 10.3%). Neither the incidence of spontaneous loss nor the incidence of therapeutic abortion differed between patients with disease flares and those with PIH.

### 3.4. Risk Factors for Fetal APOs

Table 2 reveals a comparison of clinical events as well as laboratory parameters in patients with or without composite fetal APOs. Disease flares at any time, active lupus nephritis, thrombocytopenia, LAC positivity, aCL antibody positivity, and hypocomplementemia were more likely to occur in patients with APOs. Multivariate analysis revealed that disease flares and aCL antibody positivity were risk factors for composite fetal APOs (Table 3).

Univariate analysis for respective fetal APOs was shown in Table 4. Multivariate analysis revealed that disease flares and aCL antibody positivity were risk factors for fetal loss. Disease flares and PIH were responsible for preterm birth. PIH was also the independent predictor of IUGR and fetal distress.

### 3.5. Risk Factors for Maternal APOs

The rates of disease flares, active lupus nephritis, thrombocytopenia, leukopenia, LAC positivity, aCL antibody positivity, and hypocomplementemia were higher in mothers with PIH than those without (Table 5). Multivariate analysis revealed that disease flares, thrombocytopenia, and aCL antibody positivity were independent predictors of PIH.

## 4. Discussion

Herein, we leveraged a retrospective multicenter study on planned pregnancy in women with SLE. All patients were in inactive or stable state prior to conception and followed by a multidisciplinary team of experts. In our research, two-thirds of the pregnancies ended in successful delivery without any fetal APOs and severe maternal disease flares occurred in only 0.4%. Our results showed that the rate of fetal loss was significantly decreased and the occurrence of moderate-to-severe disease flares was remarkably reduced in lupus patients who underwent planned pregnancy, although preterm births remained an important issue.

Our study showed that 4.9% of the pregnancies ended in fetal loss. In comparison, a meta-analysis by Smyth et al. including 37 studies with 1842 patients and 2751 pregnancies, whose disease activity was not strictly controlled prior to pregnancy, revealed that the rate of fetal loss was as high as 23.4% [11]. Our previous research also indicated that 28.5% of pregnancies in the general lupus patients ended in fetal loss, which was significantly higher than that in women undergoing planned pregnancy. Two prospective studies evaluating fetal outcomes in lupus patients in a stable disease state reported approximate rates of fetal loss to our study, indicating 8.4% and 4.0%, respectively [3, 12]. All the three researches, ours included, suggested that planned pregnancy was beneficial for decreasing fetal loss. The above meta-analysis also revealed a higher preterm rate in women with SLE who did not undergo planned pregnancy (39.4%) [11]. In our research, the rate of preterm birth (22.2%) exceeded that in the Chinese general population (6.2~7.2%) [13], comparable to the results from the multicenter prospective study by Clowse et al. mainly focusing on lupus patients with inactive or mild stable disease state (28.2%) [14]. In our research, most preterm births occurred after the 34th week of gestation with favorable outcomes. No neonatal death occurred. Planned pregnancy, partially by decreasing the preterm rate, could improve the overall outcomes of infants in women with SLE. PPROM is considered as the primary cause for preterm birth in the general population, followed by therapeutic or spontaneous preterm [15]. Different from the general population, in our research, therapeutic preterm was the primary cause for preterm birth. Preeclampsia and disease flares are the major causes that lead to therapeutic preterm. Therefore, disease evaluation and blood pressure monitoring is of great importance.

Major risk factors for APOs in SLE pregnancy have been investigated in multiple studies and generally fall into three categories: renal involvement, SLE disease activity, and presence of aPL antibodies [16]. In our research, risk factors for fetal APOs included disease flares during pregnancy and aCL positivity, consistent with previous findings. Our results showed that fetal loss was strongly associated with both disease flares during pregnancy and the presence of aCL. It was reported that high disease activity increased the risk of fetal loss fourfold [17]. In this study, an approximate 66.7% of pregnant patients who had fetal loss were aCL antibody-positive, compared to 9.1% in the group with live births. A previous report also pointed out that there was an increased risk in fetal loss in mothers with aCL antibodies [18]. Our study indicated that disease flares during pregnancy and PIH were responsible for preterm births in patients with SLE, consistent with previous findings [19]. A prospective research also demonstrated that a high SLEDAI score increased the possibility of preterm delivery [3]. Increase in blood pressure is associated with preterm births [20] and low offspring birth weight [21] according to previous findings. In healthy pregnant women, hypertension increases the likelihood of placenta dysfunction, resulting in fetal intrauterine distress and fetal growth restriction [22, 23]. A similar association was found in our research, showing that PIH contributed to an increased risk of IUGR and fetal distress. Overall, this data suggested that maintaining inactive disease during pregnancy, treating with positive aCL antibodies, and controlling blood pressure were important for successful pregnancy.

We observed mild–moderate flares in 21.0% of pregnancies and severe flares in 0.4%, a total of 21.4%. A multicenter prospective trial assessing maternal outcomes of pregnant women with slightly active or inactive lupus nephritis before pregnancy reported that mild-to-moderate disease flares occurred in 18.3% and severe flares in 1.4% of pregnancies, which were comparable to our findings [4]. It suggested that lupus patients undergoing planned pregnancy experienced less severe disease flares with more favorable consequences. Therefore, early recognition and prompt treatment are necessary during pregnancy and could improve disease outcomes.

In our research, the rate of PIH (11.9%) was approximately twice over the general population in China (5.2%) [24]. A retrospective study of 103 pregnancies in Chinese patients with SLE found higher frequencies of PIH (20.0%) in SLE women without planned pregnancy [25]. The PROMISSE study also demonstrated that patients with inactive disease at conception had lower rates of PIH (11.2%). In our research, the independent risk factors for PIH included disease flares during pregnancy, thrombocytopenia, and aCL positivity. Active disease was the strongest predictor of preeclampsia, consistent with previous studies [4]. A systematic review indicated that there is an association between aCL antibodies and severe preeclampsia [26].

In conclusion, our research showed that planned pregnancy improved fetal and maternal outcomes in lupus patients, presenting as lower rates of fetal loss, more favorable outcomes for preterm infants, and less severe disease flares during pregnancy. Our research reinforced the importance of planned pregnancy, which allowed women with SLE to conceive in a proper time monitored by multidisciplinary experts. Disease flares should be recognized and treated immediately in order to prevent severe complications. Blood pressure should be closely controlled during pregnancy.

## Figures and Tables

**Table 1:** Maternal and fetal outcomes in pregnant women with SLE.

Fetal adverse events	N	%
Preterm birth	54	22.2
Intrauterine growth retardation	36	14.8
Fetal distress	27	11.1
Fetal loss	12	4.9
Spontaneous abortion	1	0.4
Therapeutic abortion	5	2.1
Stillbirth	6	2.5
Neonatal death	0	0
*Maternal adverse events*		
Disease flares	52	21.4
In the first trimester	8	3.3
In the second trimester	15	6.2
In the third trimester	29	11.9
SLEPDAI max > 4		
5~9	45	18.5
10~14	6	2.5
≥15	1	0.4
Pregnancy-induced hypertension	29	11.9
Gestational hypertension	3	1.2
Preeclampsia	26	10.7
Eclampsia	0	0

**Table 2:** Association of different characteristics during pregnancy with composite fetal APOs.

Characteristics	Total (*n* = 243)	With fetal APOs (*n* = 86)	Without fetal APOs (*n* = 157)	*P* value
*Clinical manifestation at any time (n, %)*				
Flare during pregnancy	52 (21.4)	40 (46.5)	12 (7.6)	<0.001
Active lupus nephritis	45 (18.5)	32 (37.2)	13 (8.3)	<0.001
Thrombocytopenia	23 (9.5)	14 (16.3)	9 (5.7)	0.01
Leukopenia	7 (2.9)	4 (4.7)	3 (1.9)	0.2
Skin rash	19 (7.8)	9 (10.5)	10 (6.4)	0.3
Joint involvement	18 (7.4)	6 (7.0)	12 (7.6)	0.9
*Serological profile at any time (n, %)*				
Anti-dsDNA antibody positivity	93 (38.3)	34 (39.5)	59 (37.6)	0.8
Anti-Ro antibody positivity	50 (20.6)	19 (22.1)	31 (19.8)	0.7
Anti-La antibody positivity	33 (13.6)	12 (14.0)	21 (13.4)	1.0
LAC positivity	19 (7.8)	14 (16.3)	5 (3.2)	<0.001
aCL IgG positivity	25 (10.3)	21 (24.4)	4 (2.5)	<0.001
aCL IgM positivity	13 (5.3)	11 (12.8)	2(1.3)	<0.001
Anti-beta2 GP1 positivity	21 (8.6)	7 (8.1)	14 (8.9)	0.8
Hypoalbuminemia	66 (27.2)	29 (33.7)	37 (23.6)	0.09
C3 < 80 mg/dL	40 (16.5)	24 (27.9)	16 (10.2)	<0.001
C4 < 15 mg/dL	51 (21.0)	29 (33.7)	22 (14.0)	<0.001

**Table 3:** Association of different characteristics during pregnancy with APOs: results of multivariate analysis.

Characteristics	*P* value	OR	OR 95% CI
Fetal APOs			
*Composite APOs*			
Disease flares during pregnancy	<0.001	8.1	3.8–17.2
Anticardiolipin antibody positivity	<0.001	7.4	2.5–21.8
*Fetal loss*			
Disease flares during pregnancy	0.002	28.4	3.4–239.0
Anticardiolipin antibody positivity	0.004	7.8	1.9–31.4
*Preterm birth*			
Disease flares during pregnancy	0.002	3.5	1.6–7.7
PIH	<0.001	6.0	2.3–15.8
*IUGR*			
PIH	<0.001	6.7	2.9–15.8
*Fetal distress*			
PIH	<0.001	6.1	2.5–15.2
Maternal APOs			
*PIH*			
Disease flares during pregnancy	<0.001	12.2	4.4–33.3
Thrombocytopenia	0.04	4.0	1.1–14.8
aCL antibody positivity	<0.001	7.5	2.5–22.4

**Table 4:** Univariate analysis of variables associated with different fetal adverse maternal outcomes.

Characteristics	Fetal loss			Preterm birth			IUGR			Fetal distress		
	Yes	No	*P*	Yes	No	*P*	Yes	No	*P*	Yes	No	*P*
*n*	12	231	*—*	54	189	*—*	36	207	*—*	27	216	
Disease flares during pregnancy	11(91.7)	41(17.7)	<0.001	27(50.0)	25(13.2)	<0.001	16(44.4)	36(17.4)	<0.001	9(33.3)	43(19.9)	0.1
Active lupus nephritis	9(75.0)	36(15.6)	<0.001	23(42.6)	22(11.6)	<0.001	12(33.3)	33(15.9)	0.01	7(25.9)	38(17.6)	0.3
Thrombocytopenia	5(41.7)	18(7.8)	0.002	8(14.8)	15(7.9)	0.1	4(11.1)	19(9.2)	0.8	5(18.5)	18(8.3)	0.2
Leukopenia	2(16.7)	5(2.2)	0.04	2(3.7)	5(2.6)	0.7	0	7(3.4)	0.3	1(3.7)	6(2.8)	0.6
PIH	5(41.7)	24(10.4)	0.007	20(37.0)	9(4.8)	<0.001	13(36.1)	16(7.7)	<0.001	10(37.0)	19(8.8)	<0.001
Anti-dsDNA antibody positivity	5(41.7)	88(38.1)	0.8	21(38.9)	72(38.1)	1.0	16(44.4)	77(37.2)	0.4	9(33.3)	84(38.9)	0.6
Anti-Ro antibody positivity	8(66.7)	91(39.4)	0.07	21(38.9)	78(41.3)	0.8	17(47.2)	82(39.6)	0.4	16(59.3)	83(38.3)	0.04
Anti-La antibody positivity	2(16.7)	31(13.4)	0.7	8(14.8)	25(13.2)	0.8	5(13.9)	28(13.5)	1.0	3(11.1)	30(13.9)	0.7
LAC positivity	5(41.7)	14(6.1)	0.001	8(14.8)	11(5.8)	0.03	4(11.1)	15(7.2)	0.4	4(14.8)	15(6.9)	0.2
Anticardiolipin antibody positivity	8(66.7)	21(9.1)	<0.001	15(27.8)	14(7.4)	<0.001	7(25.9)	22(10.2)	0.03	9(25.0)	20(9.7)	0.02
Anti-beta2 GP1 positivity	2(16.7)	19(8.2)	0.3	4(7.4)	17(9.0)	0.7	2(5.6)	19(9.2)	0.7	2(7.4)	19(8.8)	0.8
Hypoalbuminemia	6(50.0)	60(26.0)	0.09	17(31.5)	49(25.9)	0.4	7(19.4)	59(28.5)	0.3	7(25.9)	59(27.3)	0.9
Hypocomplementemia	9(75.0)	47(20.3)	<0.001	17(31.5)	39(20.6)	0.1	10(27.8)	46(22.2)	0.3	6(22.2)	50(23.1)	0.9
Hydroxychloroquine	9(75.0)	117(50.6)	0.1	29(53.7)	97(51.3)	0.8	16(44.4)	46(22.2)	0.3	11(40.7)	115(53.)	0.2

**Table 5:** Univariate analysis of variables associated with adverse maternal outcomes.

Characteristics	PIH		
	Yes (*n* = 29)	No (*n* = 214)	*P* value
Disease flares during pregnancy	22(75.9)	30(14.0)	<0.001
Active lupus nephritis	18(62.1)	27(12.6)	<0.001
Thrombocytopenia	9(31.0)	14(6.5)	<0.001
Leukopenia	3(10.3)	4(1.9)	0.04
Anti-dsDNA antibody positivity	12(41.4)	81(37.9)	0.7
Anti-Ro antibody positivity	14(48.3)	85(39.7)	0.4
Anti-La antibody positivity	2(6.9)	31(14.5)	0.4
LAC positivity	7(24.1)	12(5.6)	0.003
Anticardiolipin antibody positivity	15(51.7)	14(6.5)	<0.001
Anti-beta2 GP1 positivity	1(3.4)	20(9.3)	0.5
Hypoalbuminemia	11(37.9)	55(25.7)	0.2
Hypocomplementemia	14(48.3)	42(19.6)	0.001
Hydroxychloroquine	13(44.8)	113(52.8)	0.4
